# Evaluation of pretreatment serum interleukin-6 and tumour necrosis factor alpha as a potential biomarker for recurrence in patients with oral squamous cell carcinoma

**DOI:** 10.4317/medoral.20373

**Published:** 2015-04-10

**Authors:** Ivana Skrinjar, Vlaho Brailo, Danica Vidovic-Juras, Vanja Vucicevic-Boras, Aleksandar Milenovic

**Affiliations:** 1 Department of Oral Medicine, Clinical Hospital Center Zagreb, Gunduliceva 5, 10000 Zagreb, Croatia; 2 Department of Oral Medicine, School of Dental Medicine, University of Zagreb, Gunduliceva 5, 10000 Zagreb, Croatia; 3 Department of Maxillofacial Surgery, University Hospital Dubrava Zagreb, Av. Gojka Suska 6, 10000 Zagreb, Croatia

## Abstract

**Background:**

Oral squamous cell carcinoma (OSCC) constitutes 3 percent of all cancers with predominant occurrence in middle aged and elderly males. Tumour recurrence worsens disease prognosis and decreases quality of life in patients with OSCC. Proinflammatory cytokines such as interleukin-6 (IL-6) and tumour necrosis factor alpha (TNF-α) have been suggested to play a certain role in variety of tumours. The aim of this study was to investigate the relationship of pretreatment serum IL-6 and TNF-α levels on tumour recurrence in patients with OSCC in order to identify potential biomarkers for the early detection of disease recurrence.

**Material and Methods:**

The patients with newly diagnosed OSCC were treated and followed from the first visit from November 2006 until January 2008. Serum IL-6 and TNF-α concentrations were measured. The records of the patients were re-examined in July 2012 and data were recorded about cancer characteristics and tumour recurrence. Disease free survival was analyzed by Kaplan-Meier survival curves, log rank test and Cox proportional hazards regression.

**Results:**

Serum IL-6 was shown as an independent risk factor for tumour recurrence.

**Conclusions:**

Pretreatment serum IL-6 concentration may be a useful biomarker for identification of OSCC patients with increased risk of the disease recurrence.

**Key words:** Serum IL-6, serum TNF-α, oral cancer, recurrence.

## Introduction

Oral squamous cell carcinoma (OSCC) accounts for 95% of all oral cavity and oropharyngeal cancers (1). Despite the improvement in surgical and radiation therapy, 5-year survival rate has not significantly improved. The stage of the tumour at the time of diagnosis is important predictor of survival. Tumour recurrence significantly worsens disease prognosis and decreases quality of life in patients with OSCC.

It has been shown that inflammation plays a key role in different stages of carcinogenesis (2). Serum concentrations of cytokines can be attributed to the secretion from tumour cells as well to systemic immune response to the tumour growth (3).

Pro inflammatory cytokines such as interleukin-6 (IL-6) and tumour necrosis factor alpha (TNF-α) have been suggested to play a certain role in the variety of tumours. The increased levels of serum IL-6 in patients with head and neck squamous cell carcinoma (HNSCC) compared to the healthy controls have been found in previous reports (4-6). Serum IL-6 concentration might correlate with the stage of tumour proliferation in patients with HNSCC (7) and can be useful in the detection of metastases (8).

It has been shown that TNF-α could act as endogenous pro motor of tumour via activation of NF-kB transcriptional factor (9). Different tumour cells produce TNF-α, including ovarian cancer and breast cancer (2). Recent study has shown that high levels of plasma TNF-α and C-reactive protein affect survival in patients with HNSCC (10).

The aim of this study was to investigate the correlation of serum IL-6 and TNF-α levels on tumour recurrence in patients with OSCC in order to identify potential biomarkers for early detection of disease recurrence.

## Material and Methods

This was a prospective study of patients with newly diagnosed OSCC. The patients were treated and followed from the first visit (November 2006 - January 2008) until June 2012 at the Department of Maxillofacial Surgery University Hospital Dubrava Zagreb, Croatia. The study included 36 patients (30 man and 6 women) with histologic ally confirmed OSCC, mean age 59 (range 37-78) and 31 healthy volunteer blood donors (19 man and 12 women) with healthy oral mucosa, mean age 58 (range 50-78). Cancer stage was determined by use of TNM classification according to American Joint Committee on Cancer. Exclusion criteria for OSCC patients was assigned diagnosis of autoimmune disease.

Prior to the study, all the participants signed informed consent and the study was performed in accordance with the Declaration of Helsinki. The study was approved by the Ethics Committee of the School of Dental Medicine University of Zagreb and financed by the Croatian Ministry of Science and Technology, project number 065-0650445-0485.

The recruitment of the patients began in November 2006 when blood samples for IL-6 and TNF-α analysis were collected. Notations were made about age, sex, smoking status and cancer site. Cancer site was divided into anterior and posterior regions of the mouth. Periodontal status was determined by Community Periodontal Index of Treatment Needs (CPITN) (11). The records were again reexamined in July 2012 and data were recorded again about cancer stage, N classification, type of treatment and local recurrence, regional recurrence and/or metastatic disease. The type of treatment was dichotomized as surgery with/without radiotherapy versus chemoradiotherapy. The patients were censored as having a recurrence or not at their last annual chart review. Time until recurrence was noticed as disease free survival.

- Blood collection and cytokines analysis 

Blood samples were collected between 8 and 10 A.M. with standard method from the antecubital veins. In patients with OSCC blood was collected before any kind of treatment. Samples were stored at room temperature for 90 minutes and then centrifuged for 10 minutes (ROTINA 35, Hettich, Germany). Sera were separated with Pasteur pipette and frozen at -70°C until analyzed.

Concentration of IL-6 and TNF-α were analyzed by chemiluminiscent enzyme-linked immune assay (ELISA).

For each cytokine 300 µL of sera was put into vial and transferred into container with marbles on which primary antibodies (IMMULITE, Siemens, Germany) specific for certain cytokine were adsorbed. After incubation and washing, secondary antibody labeled with enzyme (IMMULITE, Siemens, Germany) was added. After washing unbind secondary antibody, luminogenous substrate (adamantyl-1,2-dioxetane-phosphate) was added. Degradation of luminogenous substrate results in emission of light which is measured by luminometer. Concentrations of cytokines were written by device in pg/ml. The lower limit of detection for IL-6 was 2 pg/ml and for TNF-α 4 pg/ml. The highest limit of detection for both cytokines was 1000 pg/ml. If the concentrations were not detectable value of 0.001 pg/ml was recorded.

Elevated levels for each cytokine were defined as higher levels than the 95. percentile of a value in the control subjects (12).

- Statistical Analysis

Statistical analysis was performed by MedCalc 13.0.0. statistical programme (Ostend, Belgium). The Fisher exact test was used to compare cytokine concentrations between groups. The Mann-Whitney U test and Kruskal-Wallis test were used to determine the association of concentration of IL-6 and TNF-α with control variables.

Disease free survival was analyzed by Kaplan-Meier survival curves, log rank test and Cox proportional hazards regression. *P*-value <0.05 was considered significant in all analyses.

## Results

There were no significant differences regarding age and sex between the patients and the controls.

The elevated level of serum IL-6 was defined as >6 pg/ml and elevated level of serum TNF-α was defined as >28.6 pg/ml according to previous study described earlier (12).

The patients with OSCC had higher concentration of serum IL-6 than the controls (Fisher exact test, *p*=0.006). No difference was found in the concentration of serum TNF-α between the patients and the controls ( Table 1).  Table 2 shows clinical characteristic of the disease. Most of the patients had tumour stage 3 or stage 4, and only one patient had distant metastases in the time of the diagnosis.

**Table 1:**
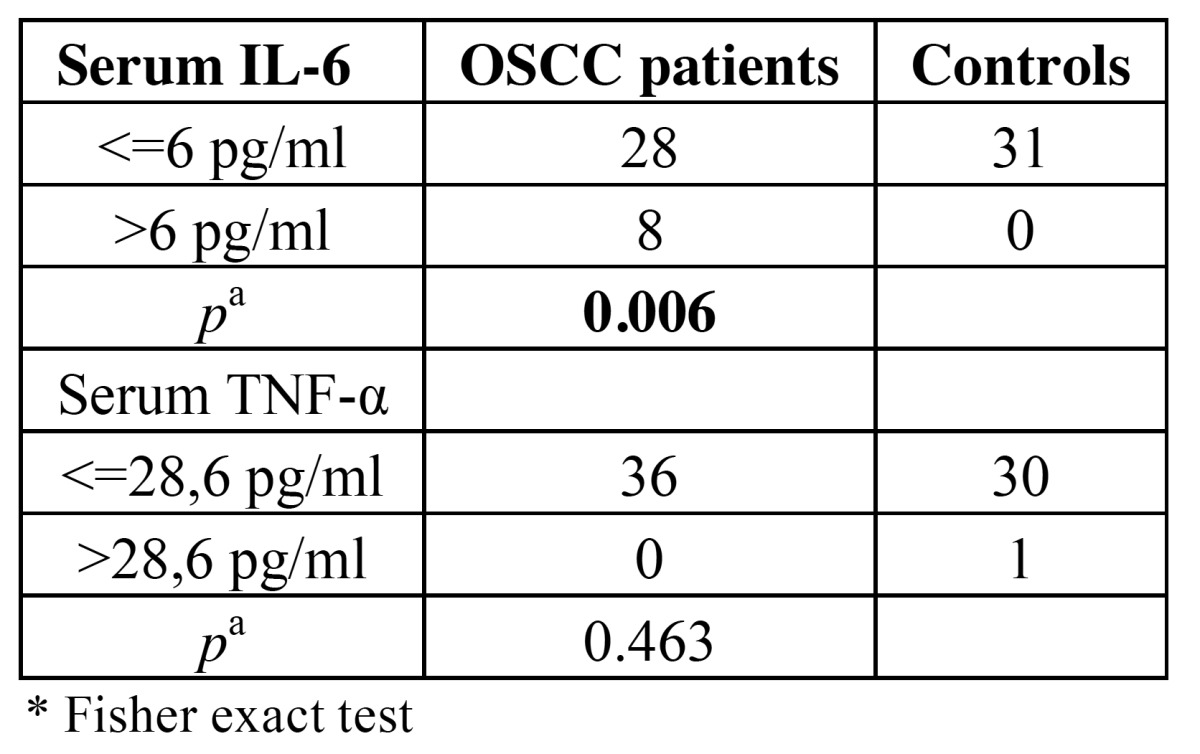
Serum IL-6 and TNF-α in oral cancer patients and control subjects.

**Table 2:**
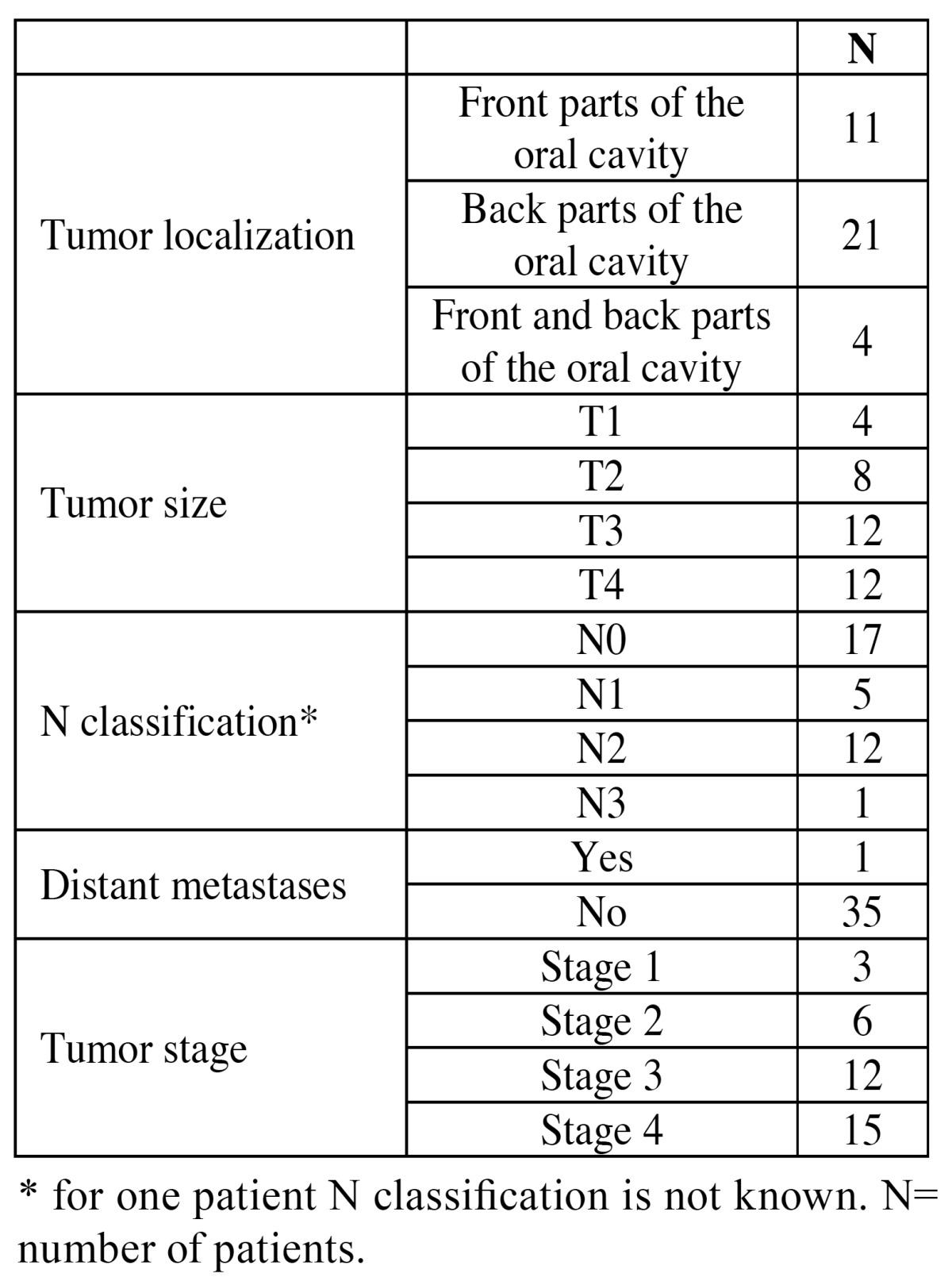
Clinical characteristics of the disease.

There were no differences between concentrations of serum IL-6 and TNF-α according to the age, cancer site, cancer stage and N classification ( Table 3). It was shown that men have significantly higher serum IL-6 and TNF-α levels when compared with the women (*p*=0.03; *p*=0.02 respectively) and that smokers have significantly higher serum TNF-α levels in comparison to the nonsmokers (*p*=0.04). Serum IL-6 concentrations did not differ between smokers and nonsmokers.

**Table 3:**
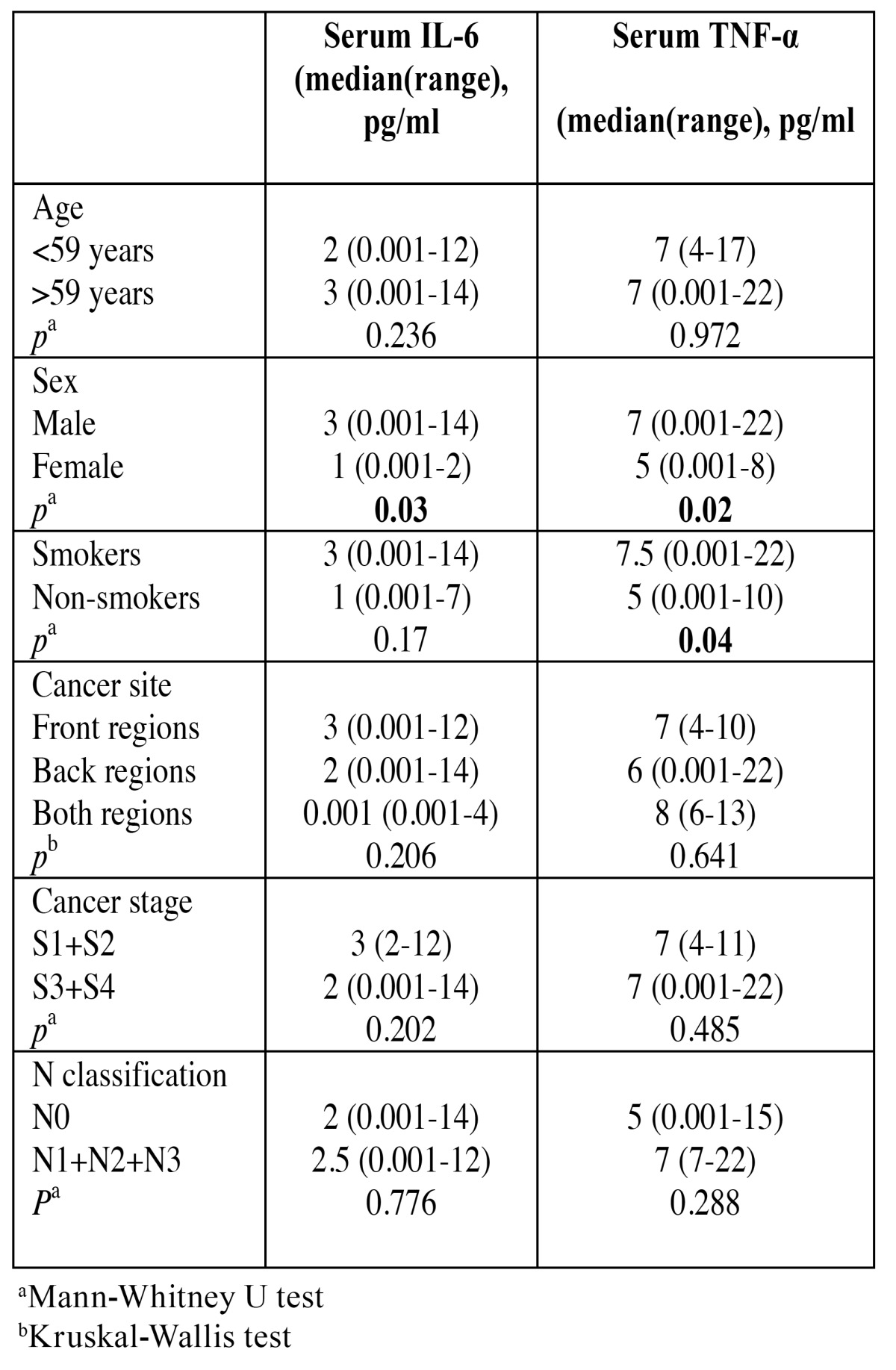
Serum IL-6 and TNF-α and contol variables.

**Figure 1:**
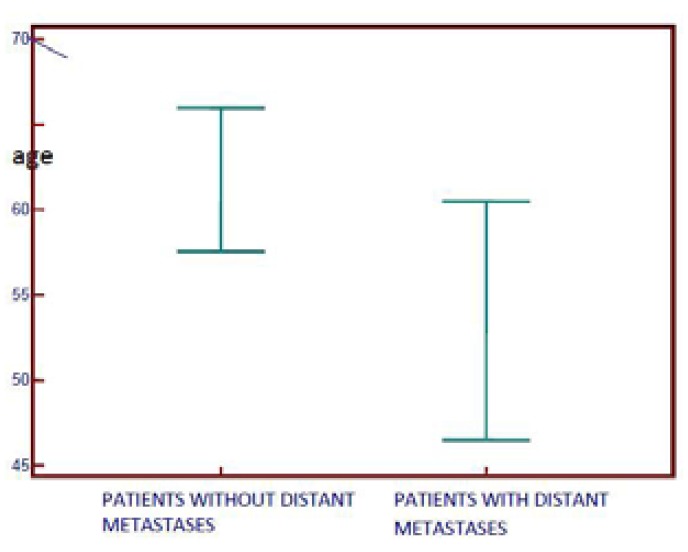
Distant metastases according to age in patients with oral squamous cell carcinoma. The median age of patients with metastases was 53, the median age of patients without metastases was 61.

The results show that patients who had distant metastases were younger (Fig. 1). Univariate analysis of tumour recurrence. Two-year recurrence rate was forty-four percent.

**Figure 2:**
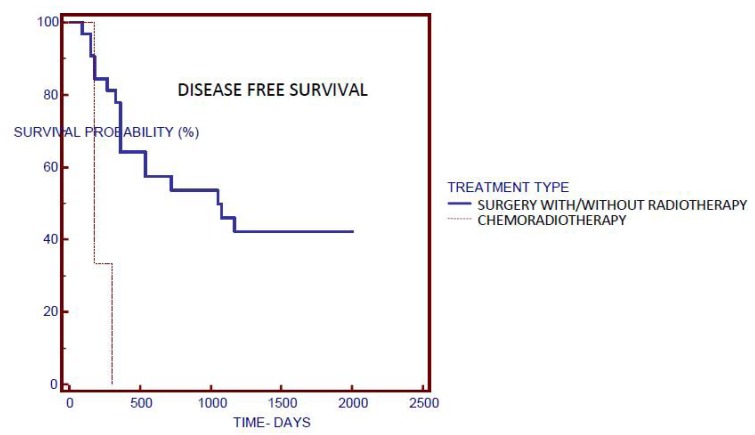
A Kaplan-Meier plot of treatment type.

Univariate analysis by Kaplan-Meier survival curves and log rank test failed to show significance between serum IL-6 or TNF-α and disease free survival. Other factors including age, sex, smoking status, cancer site, cancer stage and N classification also did not correlate significantly with disease free survival. The treatment type was shown as an independent risk factor for recurrence (Fig. 2). The patients who underwent surgery with/without radiotherapy had median disease free survival of 1080 days when compared to the patients who solely underwent chemoradiotherapy without surgery, and who had median disease free survival of 180 days.

Multivariate analysis of tumour recurrence

All variables were analyzed by multivariate analysis to determine their significance when cofactors were controlled as was recommended in the previous study of Sun *et al*. (13).

For the purpose of multivariate analysis serum IL-6 and TNF-α was log transformed to eliminate the effect of extreme values.

The results of multivariate Cox proportional hazards models for recurrence are shown in  Table 4. Serum IL-6, cancer site and type of therapy were significant risk factors for tumour recurrence. Higher pretreatment serum levels of IL-6 were significantly associated with disease recurrence (HR=1.57; 95% CI, 1.06-2.33, *p*=0.03). The patients with tumours located in the anterior and posterior regions of the mouth had higher risk for recurrence (HR=10.59; 95% CI, 2.04.-55.06; *p*=0.005). The treatment type was also shown as independent factor of recurrence as was in univariate analysis (HR=24.83; 95% CI, 4.18-147.40, *p*<0.001). Serum TNF-α, age, sex, smoking status, cancer stage and N classification were not significantly associated with the tumour recurrence.

**Table 4:**
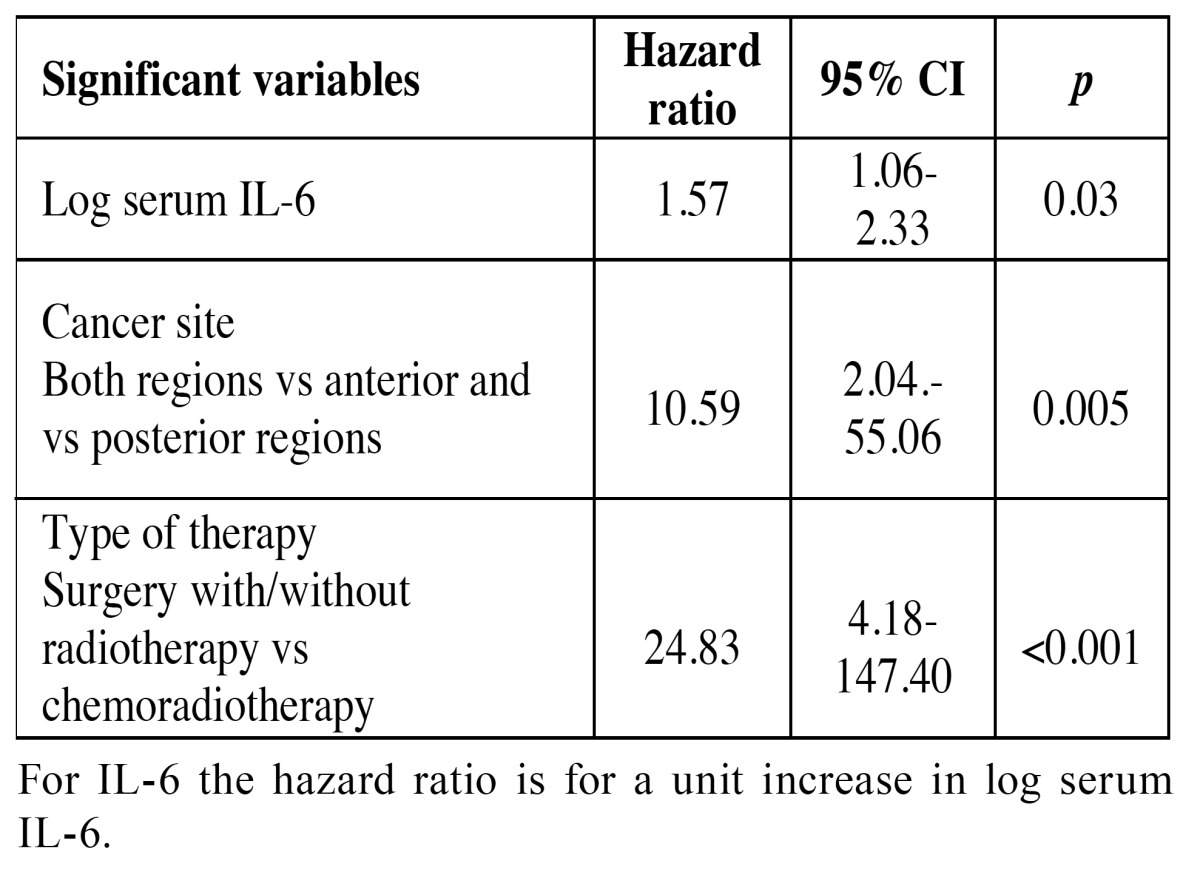
Cox proportional hazards regression models for recurrence events.

## Discussion

Our results have shown that pretreatment serum IL-6 concentration may be a useful marker for identification of OSCC patients with increased risk for the disease recurrence. Early diagnosis of loco regional or metastatic disease plays an important role in the prognosis of the patients with oral cancer (14-16). In this study, serum concentration of IL-6 was higher in oral cancer patients when compared to the controls which was also confirmed in other studies (4,6,8,17) Furthermore, the results of this study show that serum IL-6 was an independent risk factor for the disease recurrence.

Elevated serum IL-6 concentrations indicate that systemic inflammatory response is involved in carcinogenesis. Tumour growth and invasion in the surrounding tissues induces inflammation while tumour necrosis and hypoxia may activate inflammatory response. Moreover, tumour cells themselves may increase production of inflammatory cytokines such as IL-6 (18). Those cytokines react with immune and vascular system and stimulate tumour cells growth, tumour invasion and metastases (19). Some authors (10,20,21) did not report difference between serum IL-6 concentrations in patients with OSCC and controls. In the sudy of Andersson *et al*. (10) patients with head and neck squamous cell carcinoma were not age and sex matched with the healthy controls as it was in our study. The study of Vucicevic Boras *et al*. (21) had differences in age between control group (mean age 25) and patients with OSCC (mean age 55). The study of Czerninski *et al*. (20) included potentially premalignant disorders (oral dysplasia or oral lichen planus) and six healthy volunteers as a control against only healthy volunteers in our study. Those differences in methodologies could explain different results. The results of this and other studies (5,7,22) indicate that serum IL-6 is elevated in OSCC patients and may be potential marker for tumour recurrence. The patients with elevated serum IL-6 concentration may benefit from more frequent recall visits for the purpose of early diagnosis of tumour recurrence.

Although our results have shown that serum IL-6 concentration was higher in men than women, sex was not significant risk factor for tumour recurrence as was shown by other authors (14,22). Although sex did not have impact on tumour recurrence, both serum IL-6 and TNF-α were higher in men. Some authors have shown higher serum IL-6 and TNF-α levels in healthy smokers when compared to the healthy nonsmokers and higher TNF-α levels in patients with periodontitis in comparison to the healthy controls (23). The results of this study have shown that men did not smoke significantly more than women (data not shown). There were no significant differences regarding CPITN between men and women, as well as no significant difference in CPITN between OSCC patients and healthy controls (data not shown). Therefore, we can exclude smoking and periodontal disease as a potential causes of differences between IL-6 and TNF-α serum concentration between men and women. Elevated levels of IL-6 and TNF-α in men compared to women could probably be explained by the fact that we had unequal sample size regarding men and women. This result has to be confirmed or rejected in the future study with approximate equal percent of both genders.

Tumour recurrence was not dependent on the participants age which was also confirmed by others (14,24). Nevertheless, patients who had distant metastases were significantly younger which confirms the fact that oral cancer is more aggressive in younger people (25).

Cancer site was not associated with serum concentrations of IL-6 and TNF-α. However cancer site was shown as a significant risk factor for tumour recurrence. Tumours located in the anterior and posterior regions of the oral cavity had worse prognosis in comparison to those located in the anterior or posterior regions only. There were four patients within this group and all of them had tumours which involved floor of the mouth and the base of the tongue. This finding indicates that tumours located in those regions are more aggressive which could be explained by different lymphatic drainage of the anterior and posterior regions of the mouth (26). Generally, lymph from the posterior regions drains to lower cervical lymph nodes and involvement of those nodes could be associated with poor prognosis in OSCC patients.

The treatment type was shown as an independent risk factor for tumour recurrence. The patients who underwent chemoradiotherapy only had worse prognosis in comparison to the patients who underwent surgery with/without radiotherapy. Cancer stage, on the other hand was not associated with tumour recurrence. Even though the treatment is based on the stage of the disease, different treatment modalities can be applied within the same stage based on factors like tumour site, capsule perforation, patient’s general state etc. Three patients treated with chemoraditherapy had tumours localized in the posterior regions of oral cavity which could have contributed to the outcome. Given the fact that there were only three patients in this group, this result should be taken with caution.

Serum TNF-α concentration did not differ significantly between OSCC patients and healthy controls as was shown by other authors (6,27) and was not shown to be a significant risk factor for tumour recurrence. In this study, smokers had higher serum TNF-α concentrations in comparison to the nonsmokers which indicates systemic inflammatory response to the ingredients in cigarette smoke as it was shown previously (23).

In conclusion, pretreatment serum IL-6 concentration may be a useful biomarker for early detection of recurrence in patients with OSCC. Patients with elevated pretreatment serum IL-6 levels should therefore be recalled more frequently.

Further prospective studies involving greater number of OSCC patients are needed to confirm these findings.
